# Mr. Weldon's Case of Angina Pectoris

**Published:** 1806-12

**Authors:** 


### *To the Editors of the Medical and Physical Journal.*

Gentlemen,

SAMUEL FORD, a man of Negro extraction, a native of the West Indies, sixty-four years of age, during the early and middle part of his life enjoyed good health. He had been addicted to venery, otherwise he was a very temperate liver. He never had any symptom of the gout, but had, at times, been subject to rheumatism.

I first saw him in August, 1799. He had then been ill for several months with a disease of his breast, that came in paroxysms at uncertain periods.

The first attack was about eighteen months before, when on a journey from Scotland to London; and he supposed it to be produced by cold, consequent to sleeping in a damp bed, while on the road. He was able to proceed on his journey without much delay. During the first six or eight months after, he had several of these paroxysms. They generally came on while walking; and after sitting down for some minutes, they gradually went off, leaving him much agitated, and very low; but without any other particular symptoms.

They gradually became more frequent and violent, until at last they were brought on by the least hurry either of body or mind.

When I first saw him, the paroxysms succeeded each other, sometimes in twelve hours, sometimes in three or four days, but mostly in from forty to sixty hours. They came on more frequently between midnight and eight o'clock in the morning than during the day. But they were by no means confined to these hours; any little hurry brought on a paroxysm at any time. In the night, it was thought to be brought on by a sudden turn in bed during sleep. In general, however, the paroxysm appeared to come on without any immediate exciting causes, and without any previous information. They differed in violence, duration, and frequency.

A sudden and violent pain was felt under the sternum, accompanied with a sensation that was peculiar and inexpressibly distressing; producing in the mind great terror and apprehension of instant death. He called it a fulness and distension, a stoppage of breath, a sense of suffocation; but, upon close inquiry, he used these expressions for want of a proper term to express his feelings by, rather than as being exactly what he felt. Great agitation attended, with palpitation of the heart. The respiration was irregular, slow, and scarcely perceptible; but it was not suspended. In one of the paroxysms, when I happened to be present, he made, at my desire, two or three pretty full inspirations and expirations without any particular difficulty, beyond what might be expected from the distress and pain he suffered. After ten, fifteen, or twenty, sometimes even thirty minutes, the distress and pain began to abate, a pain extended over the fore-part of the chest, and from thence over one or both shoulders, and sometimes down the fore-arm. This was accompanied with a rumbling and uneasiness in the bowels and stomach, and with large discharges of wind from the mouth.

During the paroxysm the patient generally sat with his shoulders and head inclined forwards.

The frequency of these attacks at length impaired his strength very much. His digestion became imperfect, with a disposition to costiveness; the strength of his mind became much impaired; and his dread of the next succeeding paroxysm, which was always very great, became excessive.

Opening medicines, cordials, opiates, and tonics, were administered according to circumstances, but without any immediate or apparent effect at first. But, after a time, an opiate was thought to delay or prevent the fit.

After three or four months, the frequency of the paroxysms began to abate; and, after some longer time, their violence also gradually diminished; his general health at the same time was observed to be improving.

These

These favourable appearances continued, subjected to two or three slighter relapses, until, in about two years and a half, the paroxysms had nearly disappeared.

He did not recover his former good health. He had a constant disposition to costiveness, with flatulence, occasional head-achs, and other symptoms of indigestion.

He became very subject also to catarrhal complaints; but he had no serious disease until the latter end of June last, when he was attacked with a smart fever, accompanied with a dull pain in the side, an obstinate cough, and an expectoration of mucus verging towards pus. The fever gradually increased, the pulse becoming very hard, quick, frequent, and full; yielding, with great difficulty, to repeated bleeding, and soon recovering its former hardness. After these symptoms had continued for some days, they were attended with several large discharges of blood from the lungs.

By repeated bleedings and evacuations the hæmorrhages at length ceased, and the hardness of the pulse subsided, but left him in a very emaciated debilitated state, with a large expectoration, and without appetite or powers of digestion. Under these symptoms, he lingered until the 3d instant, when he died.

On examination of the cavities of the thorax, the left lung was free from disease; the cavity of its pleura was free from adhesion, except at the anterior part of the inferior lobe, where it is truncated to leave room for the heart. At this part was a firm adhesion between the surfaces of the pleura, extending near three inches in length and two in breadth, uniting the surfaces of the pleura covering the apex of the lung, the ribs and the adjoining part of the pericardium closely together. It appeared to have been of long existence. The right cavity of the chest was free from adhesion; the lung fitted the cavity, and through the greater part of its substance, was become firm and inelastic, containing but a very small portion of air. It appeared to consist of large undefined tumours, surrounded by small portions of lung that were free from disease. On cutting into its substance, the cells were found to be filled with an imperfect dark coloured fluid pus, which oozed out as from a sponge. The bronchiæ were filled with the same fluid, and a considerable quantity of it was effused into the trachea. The membrane lining the bronchiæ was very generally ulcerated; in many parts the ulcerations had extended to a considerable depth; there was was no appearance of tubercles, nor of any circumscribed cavity or abscess.

The cavity of the pericardium appeared in an healthy state, except that it contained about an ounce and a half of a tolerably transparent fluid.

The heart was very small, pretty free from adeps, its valves and substance in an healthy state. The semilunar valves of the aorta and pulmonary artery were in their natural state; the coronary vessels were free from disease; the aorta was capable of performing its functions, although not free from disease; the inner membrane was thickened in numerous spots, but there was not any bony deposit, nor any apparent disposition to aneurism.

The liver was in an healthy state; the spleen very small and sound; the œsophagus, stomach, and intestines, were free from any appearance of disease.

There happened to be an incipient hydrocele in one of the spermatic cords. It was situated a little above the upper part of the cavity of the tunica vaginalis, and behind the spermatic vessels. If this tumor had increased to a large size, the spermatic vessels would have spread over the fore-part of it, and might, without caution, have been wounded in an operation.

10, *Wigmore-Street, Oct.* 20, 1806.

